# Extracorporeal Shockwave Therapy in the Treatment of Nonunion in Long Bones: A Systematic Review and Meta-Analysis

**DOI:** 10.3390/jcm11071977

**Published:** 2022-04-01

**Authors:** Valerio Sansone, Domenico Ravier, Valerio Pascale, Rachel Applefield, Massimo Del Fabbro, Nicolò Martinelli

**Affiliations:** 1 Department of Orthopedics, IRCCS Orthopedic Institute Galeazzi, Via R. Galeazzi 4, 20100 Milan, Italy; valerio.sansone@unimi.it (V.S.); domenicoravier@gmail.com (D.R.); valerio.pascale@unimi.it (V.P.); r.applefield@gmail.com (R.A.); massimo.delfabbro@unimi.it (M.D.F.); 2 Department of Biomedical, Surgical and Dental Sciences, University of Milan, 20122 Milan, Italy

**Keywords:** pseudoarthrosis, nonunion, extracorporeal shockwave therapy, long bone, systematic review, meta-analysis

## Abstract

Background: Nonunion is one of the most challenging problems in the field of orthopedics. The aim of this study was to perform a systematic review of the literature to evaluate the effectiveness of extracorporeal shockwave therapy (ESWT) in the treatment of nonunion in long bones. Methods: We conducted a search of three databases (PubMed, Scopus, and Web of Science) and found 646 total publications, of which 23 met our inclusion criteria. Results: Out of 1200 total long bone nonunions, 876 (73%) healed after being treated with ESWT. Hypertrophic cases achieved 3-fold higher healing rates when compared to oligotrophic or atrophic cases (*p* = 0.003). Metatarsal bones were the most receptive to ESWT, achieving a healing rate of 90%, followed by tibiae (75.54%), femurs (66.9%) and humeri (63.9%). Short periods between injury and treatment lead to higher healing rates (*p* < 0.02). Conversely, 6 months of follow-up after the treatment appears to be too brief to evaluate the full healing potential of the treatment; several studies showed that healing rates continued to increase at follow-ups beyond 6 months after the last ESWT treatment (*p* < 0.01). Conclusions: ESWT is a promising approach for treating nonunions. At present, a wide range of treatment protocols are used, and more research is needed to determine which protocols are the most effective.

## 1. Introduction

Pseudoarthrosis, commonly known as nonunion, is among the most challenging pathologies in the orthopedic field. The incidence, which is estimated to be 5–10% [1], but, according to some authors [2], could be as high as 50%, varies greatly depending on the type of fracture, anatomical site, and whether the fracture site is or open or closed. However, because of improved survival rates in patients with polytrauma, the incidence is predicted to increase [3] Nonunions may cause patients long-term physical disability as well as mental health problems, with elevated economic burden [4,5].

A plethora of surgical techniques are used to treat nonunion with a success rate of approximately 80% of patients achieving good to excellent final restoration of mechanical axis alignment and proper length [6]. Nevertheless, these results included all types of nonunions, and in the case of atrophic nonunions, the success rate would be significantly lower. Furthermore, in cases requiring multiple surgeries, the healing rate drops notably. Consequentially, bone regeneration strategies have been implemented for enhancing nonunion healing. Autologous bone grafting is currently the gold standard; however, its supply is limited and its potential for repair is unpredictable [7]. Furthermore, it requires an additional surgical site and is correlated to morbidities linked to the harvesting procedure [8].

Surgery of nonunion can be a vital part of the solution; in the case of dislocated nonunions, indeed, it is a necessary component of the solution, but it can also be part of the problem if, for example, the fixation is excessively rigid or the surgeon does not achieve adequate alignment.

Aside from surgery, stem cell therapies such as percutaneous bone marrow aspirate concentrate (BMAC) transplants have been shown to be effective in the treatment of nonunions [9]. Extracorporeal shockwave therapy (ESWT) is another conservative treatment option that has been successfully used in delayed healing as well as in definite nonunions [10,11,12]. The most pertinent findings of several experimental studies showed that shockwaves (SWs) promote mesenchymal stem cell (MSC) growth and differentiation toward osteo-progenitors through transforming growth factor β1 (TGF-β1) and vascular endothelial growth factor (VEGF) induction [13,14]. Another study showed that ESWT induces adenosine-triphosphate (ATP) release and promotes MSC osteogenic differentiation via P2x7 receptors [15]. Other studies revealed that SWs may also increase the expression of additional relevant factors, such as SDF-1 [16,17,18,19], although a consensus about the ability of ESWT to regulate SDF-1 expression has not yet been reached [20]. Finally, it was discovered that SDF-1 is crucial in the homing and repopulation of MSCs in bone marrow [21].

Moreover, for stem cell migration to occur, adequate vascularity is essential [21,22], and SWs have been shown to be effective in fostering neovascularization and bone repair [20,21,22]. In animal studies, SWs amplified the thickness and volume of the treated trabecular bone [23,24]. In conclusion, ESWT is a non-invasive and effective instrument for promoting bone healing.

Regarding cases of long bone fracture nonunions treated with ESWT, the literature reports success rates ranging from 54% to 98%, depending on the anatomic location, the nature of the nonunion and the elapsed time after injury before treatment [25,26]. Generally, nonunions are classified according to the radiological aspect. A hypertrophic nonunion is characterized by a thick bone callus and is usually considered to have excessive motion but with theoretical biological potentials for healing. An oligotrophic nonunion has scarce callus formation and is considered to have excessive motion and impaired biology. An atrophic nonunion has no callus formation due to impaired vascularity with no biological capacity of healing [27].

Considering the biological potential of healing, it has been proposed that in some cases, ESWT could be as effective as surgery in achieving union of long-bone hypertrophic nonunions [28]; however, in atrophic nonunions, ESWT was reported to be markedly less successful [13,26,29].

Although ESWT can be a viable alternative to surgery, there is a lack of consensus regarding the case-specific parameters to implement during the treatments and the varying results reported in the literature.

This systematic review and meta-analysis aims to evaluate and quantify the success of ESWT in the treatment of long bone nonunions as well as to identify any potential trends regarding treatment parameters that affect the outcomes.

## 2. Materials and Methods

### 2.1. Search Strategy

The systematic review was performed according to the Preferred Reporting Items for Systematic Reviews and Meta-analyses (PRISMA) guidelines [30]. A systematic literature search of the electronic databases PubMed, Web of Science, and Scopus was performed using the key words “ESWT AND nonunion AND long bones”. The full search criteria used for each database can be found in Appendix A. The last electronic search was performed on 5 October 2020. The definition of nonunion used in this review is “a fracture that has failed to show continuity of three of four cortices after surgical or nonsurgical treatment for six or more months from the time of the fracture-related injury, or has failed to demonstrate any radiographic change (improvement) for three consecutive months, and is associated with clinical findings consistent with a fracture nonunion (an inability to bear weight on the affected extremity, pain on palpation, or motion at the fracture site for 3 to 6 months or more following the incident traumatic event or the last surgical procedure” [5].

### 2.2. Inclusion Criteria

Studies were considered eligible for inclusion in our meta-analysis if they met the following criteria: (1) patients were treated for long bone nonunions, (2) ESWT was implemented to treat the nonunion, (3) healing rates checked by an outcome measure quantifying bony union (X-ray or CT), (4) full text available in English, Italian and German, (5) randomized controlled trials and prospective and retrospective cohort studies.

### 2.3. Exclusion Criteria

Studies were excluded from meta-analysis in case of: (1) follow-up period < 6 weeks (2) less than 5 patients and (3) skeletally immature patients.

### 2.4. Selection of the Studies

Titles and abstracts were screened independently by 2 reviewers (DR and RCA) to identify possible eligible studies meeting the inclusion criteria. In the case of disagreement, a joint decision was reached by discussion with a third reviewer (VS). Publications that did not meet the selection criteria were excluded. The full text versions of all eligible articles were obtained, and the same 2 reviewers independently assessed them to check if they met all inclusion criteria. For excluded articles, reasons for exclusion were reported.

### 2.5. Data Extraction

Relevant data from included articles were extracted and analyzed by 2 independent reviewers (DR and RCA). Cases of disagreement were subject to joint evaluation until an agreement was reached. The primary outcome was radiographic evidence of fracture healing.

### 2.6. Assessment of Quality

According to the Cochrane guidelines for systematic reviews [31], the quality of each study has been evaluated using Downs and Black checklist [32]. The Downs and Black checklist consists of 27 items, with a total score of 32 for randomized trials and 30 for non-randomized studies. The items are divided in 5 sections: Reporting; External validity; Internal validity—bias; Internal validity—confounding; and Power. According to previous studies, there is a different quality level corresponding to the different scores obtained with the Downs and Black checklist [31,32]: excellent (≥26), good (20–25), fair (15–19) and poor (≤14). All included studies were assessed using Downs and Black checklist.

### 2.7. Statistical Analysis

The software “Review Manager” (RevMan V5.3, The Nordic Cochrane Centre, Copenhagen, Denmark), was used to present the study findings and combine the estimates of the effect of ESWT [33]. The Mantel–Haenszel (M–H) method was used to combine studies using a fixed effects model. The presence of statistical heterogeneity was assessed through Q and I2 statistics, a value > 50% being considered substantial heterogeneity. Forest plots were used to display the results from individual studies and pooled estimates, and *p*  <  0.05 were regarded as statistically significant.

## 3. Results

### 3.1. Study Selection Process

**Figure 1:**
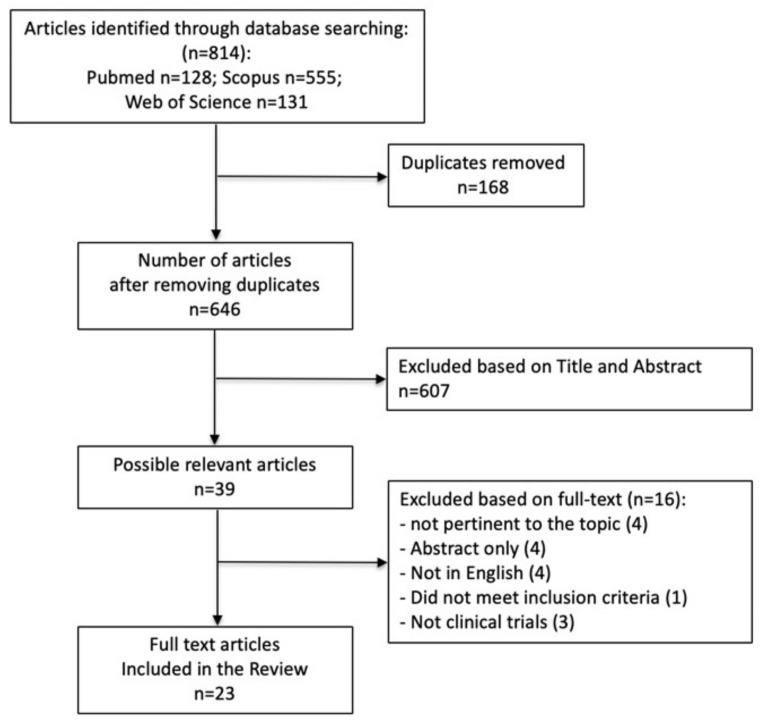
PRISMA flow diagram of the study selection process.

We used a specific search strategy to search three databases for papers (Appendix A). Figure 1 depicts a flow chart of the study selection process. Out of 646 unique publications retrieved from PubMed, Scopus, and Web of Science, 39 were included after screening the title and abstract. Out of these 39 publications, 23 met our inclusion criteria; the remainder were excluded according to the criteria listed in Figure 1.

### 3.2. Study Characteristics

Our review included two randomized controlled trials (RCTs) [28,34], one non-randomized controlled trial [35], and 20 observational studies (14 retrospective [5,9,25,26,36,37,38,39,40,41,42,43,44,45] and six prospective [11,46,47,48,49,50]). Together, the included papers reported a total of 1838 cases of delayed union or nonunion (Table 1). However, only the data for 1200 of the 1838 cases could be included in the meta-analysis, as several papers did not separate the results for long bones from those for other bones. The major characteristics of the interventions used in the 23 selected papers are summarized in Table 1. In all but one study, focused shockwaves were used. Kertzman et al. used radial shockwaves and achieved healing rates comparable to those of the focused shockwaves [5].

### 3.3. Overall Healing Rates for ESWT

Out of 1200 total long bones treated by ESWT, 876 (73%) healed after being treated with ESWT.

### 3.4. Healing Rates: Atrophic/Oligotrophic vs. Hypertrophic

**Figure 2:**
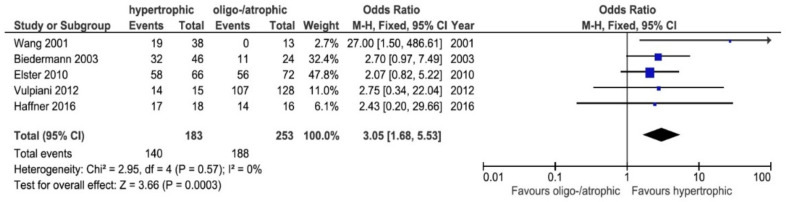
Forest plot of healing rates between oligotrophic or atrophic and hypertrophic cases (M–H, Mantel–Haenszel; CI, confidence interval) [11,26,38,43,46]. Events: number of patients with bone union after ESWT. Total: total of patients included.

Five studies reported separate results for hypertrophic and atrophic or oligotrophic cases [11,26,38,43,46]. The probability that bone healing would occur with ESWT was 3.05-fold greater in hypertrophic cases when compared to oligotrophic or atrophic cases (OR = 3.05, 95% CI: 1.68–5.53, *p* = 0.0003) (Figure 2).

### 3.5. Healing Rates by Anatomical Site

Most studies reported outcomes for all long bones together. In three studies [29,41,49], data from the outcomes of SW-treated humeri were reported separately. In total, 22 humeri were treated, 14 of which (63.6%) healed by the last follow-up. Seven studies [25,29,39,43,47,49,50] reported separate outcomes for femurs, and of 139 femurs, 93 (66.9%) healed. Eight studies [5,25,26,29,38,43,47,50] reported results for tibiae; a total of 377 tibiae were treated, of which 281 (75.54%) healed. Lastly, four studies [5,35,48,49] reported separate outcomes for metatarsals; out of a total of 50 metatarsals, 45 (90%) healed.

### 3.6. Elapsed Time between Injury and Treatment

Three studies [5,38,49] reported the time (in months) elapsed between the injury and the first ESWT treatment. In two of the three studies, a shorter time between injury was correlated to higher healing rates (*p* < 0.01) [5,37]. In the third paper, patients treated after 339 days from the injury showed an increased risk of non-healing (especially for femoral shaft and ulna) [49]. In a fourth paper, the authors did not find significant differences in the time between injury and first ESWT treatment for the “healed” and “not-healed” group [26].

### 3.7. Number of Pulses and Energy Flux Density for Femurs and Tibias

**Figure 3:**
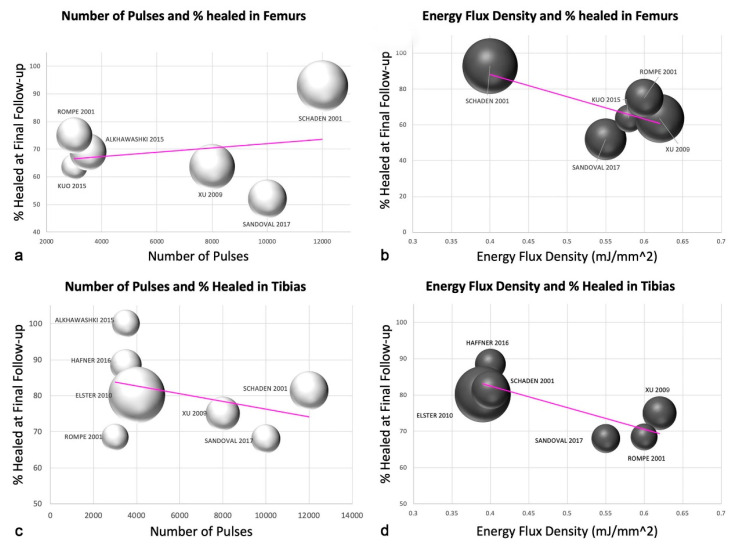
Scatter plots for the number of pulses and healing rates for femurs (**a**) and tibiae (**c**). Scatter plots for the energy flux density and healing rates in femurs (**b**) and tibiae (**d**). For Figures (**a**–**d**), the size of each plotted data point represents the number of patients in the study [25,26,29,38,39,43,44,50].

The SW parameters used in the protocols of the included studies can be found in Table 1. We did not find any correlation between the number of pulses administered to tibiae and femurs at each ESW session and the outcome (Figure 3). However, a negative trend was revealed regarding the energy flux density (EFD). Higher EFDs translated to poorer outcomes, and lower EFDs appeared to lead to higher healing rates. As a result of the small sample size, our results do not allow us to postulate on the ideal EFD to implement in treating nonunion. Nevertheless, we note that in one study of femurs treated with an EFD of 0.40 mj/mm^2^, 93% of the femurs healed [24], whereas the four other papers with evaluable data used higher EFDs, ranging from 0.55–0.62 mj/mm^2^, which corresponded to lower healing rates of 52–75% (Figure 3) [29,39,43,50]. Similarly, in tibiae, lower EFDs (0.39–0.40 mj/mm^2^) tended to correspond to higher healing rates (80–89%) (Figure 3) [39,43,50].

### 3.8. Number of ESWT Sessions

In 438 cases, it was possible to evaluate the success rate of the treatment in relation to the number of sessions carried out. Of the 177 patients who received more than one ESWT session, 136 (77.71%) healed. In the 263 patients who received only one ESWT, 224 (85.17%) healed. However, because the number of sessions were only reported as averages, we could not determine the statistical significance of this difference.

### 3.9. Time of Last Follow-Up

**Figure 4:**
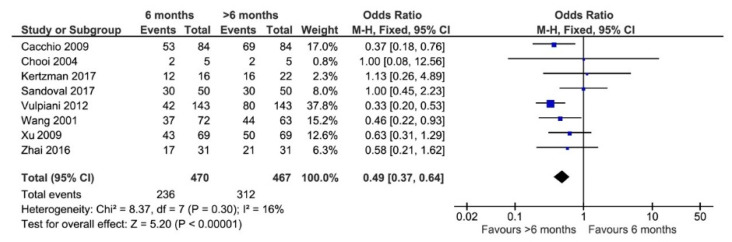
Forest plot of healing rates reported for cases at follow-ups of 6 months or longer than 6 months (M–H, Mantel–Haenszel; CI, confidence interval) [5,11,28,29,34,43,46,47]. Events: number of patients with bone union after ESWT. Total: total of patients included.

In the literature, there is no consensus regarding how long after the last ESWT the patient could still see improvement. The forest plot in Figure 4 demonstrates that 6-month follow-up was not sufficient to assess the final healing status post-ESWT. Patients that were evaluated at follow-ups beyond 6 months saw higher rates of healing than those whose last follow-up was at 6 months (OR = 0.49, 95% CI: 0.38–0.64, *p* < 0.00001). The reported data were too heterogeneous to determine the ideal time for the last follow-up; however, the mean follow-up duration for the studies in Figure 4 was 13.64 months (range 7.75–21.7 months).

### 3.10. Assessment of Quality

The average Downs and Black score for the 23 included studies was 15.2 (range 8–26), which is at the low end of the fair range. Only one study scored within the excellent range [28], and only two scored within the good range [33,34]. Ten papers had fair scores [5,26,36,37,38,39,43,46,48,49]. The remaining 10 papers had poor scores (Table 1) [11,25,29,40,41,42,44,45,47,50].

## 4. Discussion

To our knowledge, this is the first systematic review and meta-analysis to assess the effectiveness of ESWT on nonunion healing in long bones. Ideally, only randomized controlled trials would have been included in our review; however, several studies stated that ethical standards prohibited them from employing a control group. Although, some heterogeneity among studies in systematic reviews is to be expected, the inclusion or omission of results presented in the included studies in this systematic review showed exceptional variation, greatly limiting the possibility of performing consistent meta-analyses on certain aspects of ESWT. Nevertheless, with the available data, we were able to make progress towards understanding the treatment parameters that may detract from or enhance the effectiveness of ESWT in the treatment of nonunion in long bones.

The overall healing rate for nonunion in long bones was estimated to be around 73%. Although this healing rate is comparable to surgery, which is estimated to be around 80% [6], we note that our result likely underestimates the potential healing rate, as several articles reported patient dropouts while other papers included infected nonunions [26,38], which are known to exhibit very poor results with any kind of treatment if the infection is not first cured [51,52].

### 4.1. Healing Rates: Atrophic/Oligotrophic vs. Hypertrophic

Atrophic nonunions are known for being particularly difficult to treat. It had been assumed that atrophic nonunions were more resistant to treatment because of a lack of vascularization; however, in 2002, a paper by Reed et al. [53] found no basis for this rationale. In fact, their paper showed that in terms of vascularization, there was no difference between atrophic and hypertrophic nonunions. Another paper reported that in atrophic nonunion, the number of activated mesenchymal stem cells was significantly lower than in hypertrophic nonunion, which could explain why ESWT was less effective in atrophic nonunion [54]. A literature review by Zelle et al. found 10 clinical studies with patients who had delayed union or nonunions [55]. Their overall analysis of the included studies showed an overall healing rate of 76% (95% confidence interval: 73–79%). Interestingly, the difference in bony consolidation between atrophic and hypertrophic nonunions was significant, ranging from 29% for atrophic (9 of 31) to 76% for hypertrophic (185 of 243) nonunions. In this systematic review, for long bones, hypertrophic nonunions had a better chance of healing with ESWT than atrophic/oligotrophic nonunions.

### 4.2. Energy Flux Density

Different studies showed how high-energy shock waves are able to induce new bone formation in physiological as well as acutely fractured and pseudarthrotic bone [13,14]. Involvement of different growth factors such as tumor growth factor (TGF-1), various bone morphogenetic proteins (BMP) and vascular endothelial growth factor (VEGF-a), or secretion of neurotransmitter substance P has been shown [13,14]. Dose-dependent bone formation was observed in an animal model by Tischer et al. after shock wave application, with a minimum threshold energy necessary to effect bone cell formation of 0.5 mJ/mm^2^ [56]. Gollwitzer showed in rabbits new bone formation by radial ESWT, applying low energy flux densities (0.16 mJ/mm^2^) with relatively high impulse numbers (2 × 4000 impulses) [57]. However, the optimal energy regimen is still controversial. Koolen et al. have shown that a single treatment with unfocused ESW of 0.3 mJ/mm^2^ energy flux did not result in increased bone mineral content or bone mineral density of the forearm in postmenopausal women [58]. Interestingly, in this systematic review, higher healing rates were associated with lower EFD. Further studies are needed to ascertain which EFD (high-energy vs. low-energy) promotes better bone formation in nonunions.

### 4.3. Healing Rates by Anatomical Site

Although the average healing rate for all long bones in our review was 73%, the rate of healing varied greatly depending on the anatomical site. Nonunions of the metatarsals showed the highest rate of healing at 90%, followed by the tibia (75.54%), femur (66.91%), and humerus (63.64%). There are two possible rationales that could explain the disparity in success rates.

The first could be explained by the fact that the soft tissues covering bones dampen the shockwaves before they can reach the bones. Therefore, superficial bones, such as metatarsals, which have a thinner soft tissue layer through which the shockwaves must pass, may receive more energy. The second reason is based on the Hopkins effect, which states that each time a shockwave passes from a low-density material to a higher-density material, part of the shockwave is reflected [59]. When shockwaves pass through the first cortical layer and the cancellous bone, some are then reflected against the opposing cortical bone, and in some cases, these reflected waves encounter antithetical waves, thereby amplifying them [59]. This effect is more likely to apply in metatarsal bones, because the thinner cortical bone and the shorter distance between the two cortical surfaces facilitate this process [60]. Furthermore, immobilization, which can improve the healing process, is technically easier at the midfoot and tibia than at the femur and especially at the humerus.

### 4.4. Elapsed Time between Injury and Treatment

Our results showed that there is a higher success rate if the treatment is implemented closer to the time of injury (rather than later). Thus, it seems advisable to propose ESWT treatment as soon as there is evidence that the fracture is not healing properly, without waiting 6 or more months, especially in patients that have multiples risk factors for nonunion (e.g., diabetes, Cushing syndrome, corticosteroid therapy, smokers, etc.). Previous studies reported that shockwaves seems to have the potential of promoting bone healing and thus reducing the rate of nonunion in acute high-energy fractures of the lower extremities [61].

### 4.5. Risk of Bias

One potential source of bias is derived from the variation in patient characteristics. Of note, in two papers, patients with infections were included [26,38]. It is widely accepted that these cases are more difficult to treat, and their inclusion in our meta-analysis will have a negative impact on the overall healing rate.

### 4.6. Limitations

We note two main limitations in the included studies. First, in seven papers [29,35,37,39,40,41,46], the authors did not distinguish between delayed union and nonunion when describing the patient population. In several other papers, the patient population was clear in the material and methods section, but data from delayed and nonunion data were reported together. Second, we could not ascertain the ideal dose and formulation for ESWT as a result of incomplete data reporting.

Nonunion remains one of the most challenging pathologies in the field of orthopedics, with the most stubborn cases often requiring multiple surgical interventions. Thus, new treatments with the potential to improve the healing rates of nonunions should be considered. ESWT is a non-invasive alternative to surgery that has been used as a novel therapeutic method for patients with nonunions, with growing numbers of clinical studies in recent years.

## 5. Conclusions

This review demonstrated that extracorporeal shockwave therapy is a promising approach to successfully treat nonunions. The healing rates achieved with ESWT are comparable to those of surgery but do not carry the risk of possible complications. Orthopedic practitioners should consider ESWT as a viable alternative to surgery in the treatment of nonunion.

## Figures and Tables

**Table 1:** ESWT protocol, patient characteristics, and risk of bias of included studies. EFD = energy flux density, NR = not reported, NU = nonunion, DU = delayed union, * 3000–4000 pulses applied to femur, tibia, fibula, humerus; 2000–3000 pulses applied to other, smaller bones, ** 6000–10,000 pulses applied to femur and tibia; 4000 pulses applied to humerus; 3000 pulses applied to radius and ulna, 6000 pulses applied to femur and tibia; 3000 pulses applied to humerus; 2000 pulses applied to radius and ulna; 1000 pulses applied to MT, ^#^ 0.4 (group 1)–0.7 (group 2), ^◊^ 0.56 for humerus, radius, ulna; 0.62 for femur and tibia; ^⌘^ 0.47 for MT; 0.56 for humerus, radius, ulna; 0.62 for femur and tibia.

	ESWT Protocol					Patient Characteristics			Risk of Bias
Study	Type of Shock Waves	EFD (mJ/mm^2^)	Number of Pulses	Average and (Min–Max) ^#^ of Treatments	Last Follow-Up Mean (Months)	*n* Cases Treated by ESWT	*n* Long Bones	Pathology	Downs–Black Criteria
Kertzman et al., 2017	radial	0.18	3000	3.6 (2–6)	N.R.	22	20	NU	16
Sandoval et al., 2017	focused	0.55	10,000	2.8 (2–3)	12	50	50	NU	16
Haffner et al., 2016	NR	0.4	3000–4000	NR	6	58	58	NU	16
Zhai et al., 2016	focused	0.7	2900	4.8 (4–5)	18	31	31	NU	22
Alkhawashki et al., 2015	focused	NR	2000–4000 *	1.3 (1–3)	21	49	45	NU	12
Kuo et al., 2015	focused	0.58	3000	1 (1)	12	22	22	NU	16
Czarnowska-cubala et al., 2013	NR	NR	3000	NR	6	31	31	DU/NU	16
Vulpiani et al., 2012	focused	0.25–0.84	2500–3000	NR	12	143	126	NU	18
Alvarez et al., 2011	focused	0.22–0.51	2000	1 (1)	12	34	34	NU	18
Stojadinovic et al., 2011	NR	NR	NR	NR	6	349	269	DU/NU	18
Elster et al., 2010	focused	0.38–0.40	4000	1.3 (1–4)	24.7	192	192	NU	16
Furia et al., 2010	focused	0.35	2000–4000	1	64.7	23	23	NU	20
Moretti et al., 2009	NR	0.22–1.10	4000	NR	2.25	204	204	NU	9
.Cacchio et al., 2009	NR	0.4; 0.7 ^#^	4000	4	21.7	84	126	NU	26
Wang et al., 2009	focused	0.62	6000	1 (1)	15.24	42	42	NU	18
Xu et al., 2009	focused	0.56; 0.62 ^◊^	3000–10,000 **	1–2	N.R.	69	69	NU	14
Chooi et al., 2004	focused	NR	4000	1 (1)	7.75	5	5	NU	14
Bidermann et al., 2003	NR	0.7	2900	1.2 (1–2)	N.R.	70	58	DU/NU	12
Rompe et al., 2001	NR	0.6	3000	NR	9	43	43	NU	12
Schaden et al., 2001	NR	0.4	12,000	1 (1)	18	115	89	DU/NU	8
Wang et al., 2001	focused	0.47, 0.56; 0.62 ^⌘^	1000–6000	NR	12	72	72	NU	12
Vogel et al., 1997	NR	0.6	3000	NR	N.R.	48	48	NU	12
Valchanou et al., 1991	focused	NR	1000–4000	1 (1)	N.R.	82	NR	NU	8

## Appendix A. Search Strategies for Literature Searches

**PubMed:** ((ESWT OR extracorporeal shockwave therapy OR Shockwave OR shock wave OR shock waves OR shockwaves) AND (pseudarthrosis OR pseudoarthroses OR nonunion OR bone disorders OR bone fracture OR bone complications OR bone healing) AND (tibia OR femur OR humerus OR long bones)).

**Web of Science:** (TS=(ESWT OR extracorporeal shockwave therapy OR Shockwave OR shock wave OR shock waves OR shockwaves) AND TS=(pseudarthrosis OR pseudoarthroses OR nonunion OR bone disorders OR bone fracture OR bone complications OR bone healing) AND TS=(tibia OR femur OR humerus OR long bones)).

**Scopus:** (TITLE-ABS-KEY(Eswt OR shockwave OR shock AND wave OR shock AND waves OR shockwaves) AND (pseudarthrosis OR pseudoarthroses OR nonunion OR bone disorders OR bone fracture OR bone complications OR bone healing) AND (tibia OR femur OR humerus OR long bones)).
